# Case Report: Low pressure hyperbaric oxygen therapy as a potential alternative treatment for chronic inflammatory response syndrome: a case study with notable improvements in fatigue, cognition, and testing

**DOI:** 10.3389/fimmu.2025.1564788

**Published:** 2025-08-05

**Authors:** Kristen L. Coletti Giesler

**Affiliations:** Be-Well Medicine Limited Liability Corporation (LLC), Kenai, AK, United States

**Keywords:** chronic inflammatory response syndrome, fatigue, cognition impairment, cytokines, low dive hyperbaric oxygen

## Abstract

Chronic Inflammatory Response Syndrome (CIRS), a complex condition triggered by environmental exposures such as mold toxins, remains challenging to treat effectively. This case study documents the incidental findings of hyperbaric oxygen therapy (HBOT) as a potential therapeutic intervention for CIRS. A 60-year-old female patient with persistent symptoms and abnormal laboratory markers underwent 40 shallow dive HBOT sessions over 10 weeks. Clinical evaluations included symptom scoring, visual contrast sensitivity (VCS) testing, and biomarker analysis, with pre-treatment tests conducted via Quest Diagnostics and post-treatment tests via LabCorp. Results demonstrated significant improvements: resolution of all 22 reported symptoms, normalization of VCS scores (from 68% to 93%), and notable reductions in inflammatory biomarkers, including TGF-β1 and MMP-9. This case illustrates HBOT’s ability to modulate systemic inflammation, improve neurocognitive outcomes, and enhance recovery in patients with complex environmental illnesses. While limited to a single-patient design, this study underscores the need for extensive quantitative research to validate HBOT’s efficacy in managing CIRS and similar conditions.

## Introduction

Chronic Inflammatory Response Syndrome (CIRS) is a debilitating condition characterized by systemic inflammation, immune dysfunction, and neurocognitive impairment triggered by biotoxins, including mold toxins. First described by Dr. Ritchie Shoemaker, CIRS has since become a recognized multi-system, multi-symptom illness (1–3). Despite advancements in diagnostic frameworks, treatment options remain limited and are often poorly tolerated by sensitive individuals.

The patient described in this report exhibited extreme sensitivity to conventional treatments such as binders, antifungals, and detoxification protocols outlined by Nathan (4) and Crista (5). Despite attempts with antifungal medications like nystatin, itraconazole, and fluconazole, the patient experienced significant adverse effects and no symptom resolution. Hyperbaric oxygen therapy (HBOT) was introduced as an alternative therapeutic approach due to its demonstrated anti-inflammatory, neuroprotective, and systemic healing properties in neuroinflammatory and toxic injury conditions (6–8). This case adds to emerging evidence highlighting HBOT’s potential to address refractory CIRS by targeting unresolved inflammation and enhancing systemic recovery.

## Case description

### Patient information

The patient was a 60-year-old female with a history of prolonged environmental exposure to mold in a water-damaged building. Her presenting symptoms included severe fatigue, cognitive dysfunction, joint pain, and chronic headaches, which collectively impaired her daily functioning and quality of life.

NeuroQuant imaging revealed volumetric changes consistent with CIRS, including bilateral volume loss of the globus pallidum (2nd and 4th percentiles, left and right) and asymmetry in the posterior superior temporal sulcus without signal abnormalities. Additionally, a likely calcified meningioma was identified in the frontal lobe, with no underlying signal abnormality.

The patient sought care from a neurologist specializing in environmentally acquired illnesses, who confirmed the CIRS diagnosis and recommended supportive therapies.

### Past interventions and outcomes

The patient’s initial treatment plan included binders (cholestyramine, activated charcoal, clay, chlorella, saccharomyces) antifungal medications (nystatin, itraconazole, and fluconazole), and methylation nutritional support (B vitamins, magnesium, and zinc). However, she was unable to tolerate these therapies due to hypersensitivity reactions, and symptoms persisted despite adherence to the prescribed protocols (9). The lack of therapeutic progress prompted the consideration of HBOT as an alternative approach.

### Physical examination and laboratory findings

Physical examination revealed systemic signs of inflammation, including joint tenderness, cognitive impairments noted during clinical assessment and reduced physical stamina.

Laboratory analyses showed elevated inflammatory biomarkers. Visual contrast sensitivity (VCS) testing indicated biotoxin exposure, with an initial score of 68%, and TGF-β1 and MMP-9 levels were significantly elevated.

### Testing and reference ranges

## Materials and methods

### Study design

This single-patient observational study followed Shoemaker’s diagnostic framework for CIRS (10). The inclusion criteria included: the presence of at least eight symptoms across multiple domains, an abnormal VCS test, and elevated laboratory markers indicative of neuroinflammation and immune dysfunction.

### Intervention

**Figure 1:**
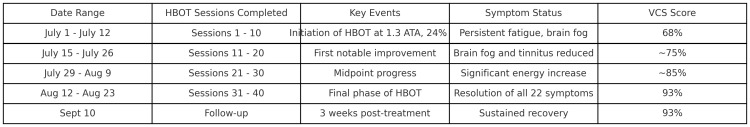
Treatment timeline and outcomes.

The progression of symptom improvement and VCS score normalization throughout the 10-week course of HBOT is illustrated in **Figure 1**. The patient engaged in an intensive therapeutic regimen comprising 40 sessions of shallow dive hyperbaric oxygen therapy (HBOT) delivered over a structured 10-week schedule. Each session was meticulously conducted in-office at Be-Well Medicine, utilizing a controlled environment with 1.3 ATA pressure and 24% oxygen. Changes in inflammatory and neuroimmune biomarkers before and after HBOT are presented in **Table 1**. The 90-minute sessions were rigorously overseen by experienced medical professionals to ensure safety and efficacy. As shown in **Table 2**, symptom scores and VCS improved markedly following HBOT.

**Table 1:** Inflammatory biomarkers pre- and post-HBOT.

Marker	Quest reference range (Pre)	LabCorp reference range (Post)
TGF-β1 (pg/mL)	<2,380	<22,306
VEGF (pg/mL)	31–86	31–86
MSH (pg/mL)	35–81	35–81
MMP-9 (ng/mL)	85–332	85–332
C3a (ng/mL)	55–486	<273.6
C4a (ng/mL)	0–2,830	<2,025.9
PAI-1 (ng/mL)	4–43	4–43

**Table 2:** CIRS symptom scoring.

Metric	Pre-Treatment (1/12/2021)	Post-Treatment (9/13/2024)
Total Symptoms	22	0
VCS Score	68%	93%

### Diagnostic assessment

Diagnosis of Chronic Inflammatory Response Syndrome (CIRS) followed Shoemaker’s criteria, incorporating:

- Detailed symptom inventory (fatigue, cognitive issues, tinnitus, joint pain).
- MARCoNS testing was performed using a nasal swab and analyzed by Microbiology Dx, a CLIA-certified laboratory specializing in nasal microbiota.” Exclusion of differential diagnoses (thyroid, rheumatologic, psychiatric disorders).
- Failure to respond to standard binders and antifungals.

## Results

### Laboratory results

This timeline illustrates the 8-week course of mild hyperbaric oxygen therapy (1.3 ATA, 24% oxygen), with symptom resolution milestones and visual contrast sensitivity (VCS) score improvement across the treatment duration.

### Patient perspective

“By session 20 I noticed my brain fog lifting … by session 40 all 22 symptoms had resolved. I felt like myself again—energized, clear-headed, and hopeful in a way I hadn’t in years.”

## Discussion

Much of the current literature on hyperbaric oxygen therapy (HBOT) focuses on high-pressure protocols involving hard-shell chambers delivering 100% oxygen at 2.0 to 2.8 ATA. These approaches have documented therapeutic value but can pose risks for sensitive populations, including oxidative stress, barotrauma, and neurologic side effects. **Table 3** outlines the pre- and post-treatment biomarker values showing the clinical shift. The use of hard-shell chambers delivering 100% oxygen at pressures of 2.0 to 2.8 ATA. While these high-pressure protocols have shown clinical efficacy, they may not be suitable for patients with environmental sensitivities or multiple chemical intolerances due to increased oxidative stress, risk of barotrauma, and potential for adverse neurological effects.

**Table 3:** Biomarker shifts with interpretation.

Marker	Pre-Treatment (Quest)	Quest Range	Post-Treatment (LabCorp)	LabCorp Range	Change
TGF-β1(pg/mL)	10,313	<2,380	12,869	<22,306	↓
VEGF (pg/mL)	20.6	31–86	40.0	31–86	↑
MSH (pg/mL)	2.4	35–81	13.0	35–81	↑
MMP-9 (ng/mL)	920	85–332	354	85–332	↓
C3a (ng/mL)	611	55–486	174.5	<273.6	↓
C4a (ng/mL)	1,948.7	0–2,830	611	<2,025.9	↓
PAI-1 (ng/mL)	22.4	4–43	11.0	4–43	↓

In contrast, this case employed mild hyperbaric oxygen therapy (mHBOT) at 1.3 ATA with 24% oxygen—a “low-dive” approach commonly used in functional and integrative medical settings. This gentler protocol remains underrepresented in large-scale clinical trials but has shown promise in modulating neuroinflammation, improving mitochondrial function, and enhancing cognitive outcomes in vulnerable populations. Studies such as Liu et al. (8) and Harch et al. (7) have documented biological benefits from lower-pressure HBOT, supporting its consideration in cases of chronic inflammatory or neurotoxic conditions.

This case highlights the potential of HBOT as a transformative therapy for refractory CIRS. The resolution of symptoms, normalization of VCS scores, and improvements in inflammatory biomarkers align with existing literature demonstrating HBOT’s anti-inflammatory and neuroprotective effects (8, 11, 12). Notably, the reduction in TGF-β1 and MMP-9 levels reflects systemic modulation of inflammatory pathways, which likely contributed to the patient’s recovery. Furthermore, the observed increase in VEGF and MSH levels is particularly significant. A rise in VEGF suggests enhanced angiogenesis and improved vascular function, while the rise in MSH indicates potential neuroregenerative processes and brain healing, underscoring HBOT’s capacity to address neuroinflammatory conditions comprehensively. Additionally, studies have shown that HBOT protocols can enhance cognitive function in older adults, further supporting its neuroprotective role (13). A timeline of the patient’s HBOT sessions and symptom progression is summarized in **Table 4**.

**Table 4:** Patient HBOT progression: July-September 2024.

Date Range	HBOT Sessions Completed	Key Events	Symptom Status	VCS Score
July 1 - July 12	Sessions 1 - 10	Initiation of HBOT at 1.3 ATA, 24% O2	Persistent fatigue, brain fog	68%
July 15 - July 26	Sessions 11 - 20	First notable improvement	Brain fog and tinnitus reduced	~75%
July 29 - Aug 9	Sessions 21 - 30	Midpoint progress	Significant energy increase	~85%
Aug 12 - Aug 23	Sessions 31 - 40	Final phase of HBOT	Resolution of all 22 symptoms	93%
Sept 10	Follow-up assessment	3 weeks post-treatment	Sustained recovery	93%

This patient’s complete resolution of symptoms and biomarker normalization after 40 sessions of low-pressure HBOT underscores the potential of mHBOT as a safer, well-tolerated therapeutic strategy for CIRS and warrants further clinical investigation.

### Strengths and limitations

This study’s strengths include its comprehensive assessment of clinical, laboratory, and imaging outcomes. The case provides detailed documentation of symptom resolution, biomarker shifts, and patient-reported recovery over time. However, the observational design involving a single subject limits generalizability, and the absence of a control group precludes definitive causal conclusions. Outcomes are based on a combination of subjective reports and objective laboratory data, without the benefit of blinding or long-term follow-up. Future controlled studies are needed to validate these findings, assess durability of response, and establish standardized protocols for mHBOT in CIRS management.

### Takeaway lessons

HBOT may offer a safe and effective therapeutic option for patients with refractory CIRS. This case underscores the need for larger-scale, controlled trials to explore its efficacy further and refine treatment protocols.

## Conclusion

This case underscores the transformative potential of hyperbaric oxygen therapy (HBOT) as an innovative treatment modality for Chronic Inflammatory Response Syndrome (CIRS), particularly for patients who have exhibited resistance or hypersensitivity to conventional therapeutic interventions. The observed resolution of symptoms, combined with marked improvements in neurovascular function and systemic inflammation, highlights the capacity of HBOT to address the multifaceted pathophysiology of CIRS. These findings not only reinforce the therapeutic promise of HBOT but also call for rigorous, large-scale clinical trials to validate its efficacy and optimize its application. Furthermore, the implications extend beyond CIRS, suggesting a broader relevance of HBOT in treating neuroinflammatory and environmentally acquired conditions, thereby paving the way for a paradigm shift in managing these complex illnesses.

## Data Availability

The raw data supporting the conclusions of this article will be made available by the authors, without undue reservation.
